# Superficial fabrication of gold nanoparticles modified CuO nanowires electrode for non-enzymatic glucose detection†

**DOI:** 10.1039/c8ra07516f

**Published:** 2019-01-14

**Authors:** Ashwini Kumar Mishra, Bratindranath Mukherjee, Amit Kumar, Deepak Kumar Jarwal, Smrity Ratan, Chandan Kumar, Satyabrata Jit

**Affiliations:** Department of Electronics Engineering, Indian Institute of Technology (BHU) Varanasi-221005 India sjit.ece@iitbhu.ac.in; Department of Metallurgical Engineering, Indian Institute of Technology (BHU) Varanasi-221005 India

## Abstract

This paper describes a low-cost facile method to construct gold (Au) nanoparticles (NPs) modified copper oxide (CuO) nanowires (NWs) electrode on copper foil for the detection of glucose. Copper foil has been converted to aligned CuO NWs arrays by sequential formation of Cu(OH)_2_ followed by heat treatment induced phase transformation to CuO. Au NPs are deposited on CuO NWs *via* simple reductive solution chemistry to impart high surface to volume ratio and enhanced catalytic activity of the resulting electrode. Structure, microstructure and morphology of Cu, Cu(OH)_2_ NWs, CuO NWs, and Au NPs modified CuO NWs are investigated by X-ray diffraction (XRD) and scanning electron microscopy (SEM). The homogeneous distribution of Au NPs (average diameter ∼12 nm) on CuO NWs (average diameter 100 nm and aspect ratio ∼20) is confirmed by high-resolution transmission electron microscopy (HRTEM), scanning transmission electron microscopy (STEM) and elemental mapping. This CuO based glucose detection method gives the highest sensitivity along with the maximum linearity range. This non-enzymatic glucose sensor based on Au modified CuO NWs electrode gives broad linearity range from 0.5 μM to 5.9 mM. The sensor exhibits sensitivity of 4398.8 μA mM^−1^ cm^−2^, lower detection limit of 0.5 μM, and very fast response time of ∼5 s. Properties of the proposed glucose sensor are also investigated in human blood and it is found that the sensor is highly accurate and reliable. In addition, higher sensitivity and lower detection limit confirm that this device is suitable for invasive detection in saliva and urine.

## Abstract 2

This paper describes a low-cost facile method to construct gold (Au) nanoparticles (NPs) modified copper oxide (CuO) nanowires (NWs) electrode on copper foil for the detection of glucose.

## Graphical abstract

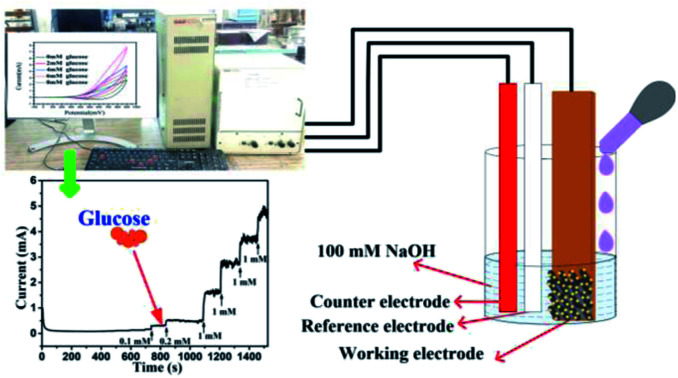

## Introduction

Diabetes is considered to be one of the most severe health problems today. It is a staid chronic disease that occurs when the pancreas does not produce sufficient amount of insulin or produces more insulin than our body requires.^1–3^ According to the World Health Organization (WHO), about 422 million adults were suffering from diabetes in 2014, which is about 8.5% of total adult population.^2^ Diabetes can lead to many complications such as heart attack, stroke, kidney failure, vision loss, and nerve damage.^2^ Although diabetes can be easily identified by measuring glucose level in the blood, a trustworthy, accurate, and fast estimation of glucose amount is a realistic challenge in biological and clinical analysis.^4,5^ Electronic sensors are considered to be effective tools for detection of various chemical analytes, such as glucose, cholesterol, fluids, gases, *etc.*^6–11^ Sensors for glucose detection take advantage of different techniques, such as electrochemistry, fluorimetry, surface plasmon resonance, and capillary-zone electrophoresis, which are commonly reported in the literature for the evaluation of blood glucose level.^12–14^ Among these techniques, electrochemistry based glucose level estimation has drawn a great deal of attention owing to its inherent simplicity, good selectivity, high sensitivity, and convenience of operation.^15–18^

Electrochemical glucose sensors are divided into two groups based on the electrocatalyst used in sensor electrodes.^9^ The first group is based on enzymes, such as GOx, GDH, *etc.*, whereas the second group is based on non-enzymatic electrocatalysts.^9^ The enzyme-based glucose sensor was first developed by Leland C. Clark, who is also called the ‘Father of Biosensors’.^19^ These enzyme-based glucose sensors exhibit high selectivity and sensitivity, and low detection limit.^20^ However, stability of the enzyme-based glucose sensors is significantly affected by the working temperature, relative humidity and the pH value.^9^ Further, the fabrication cost of such sensors is higher than that of the non-enzyme based glucose sensors.^21–24^ Owing to the aforementioned drawbacks of the enzyme-based glucose sensors, many attempts have been made to develop non-enzymatic based glucose sensors.^10,25–27^ The sensing mechanisms of non-enzyme based glucose sensors are entirely different from those of the enzyme-based sensors where electrocatalysts are required to satisfy some important requirements, such as large specific surface, outstanding conductivity, high electrocatalytic activity, effective electron transfer from electrocatalysts to the conductive substrate, good selectivity, high stability and good reproducibility.^3^

Noble metals (Pt, Au, Pd) and their alloys (Pt–Pd, Pt–Au, Au–Pd) have been explored as efficient glucose sensing materials.^28–33^ However, high cost of these noble metals limits their practical use as glucose sensors.^30,34^ For the low-cost fabrication and glucose sensing applications researchers have investigated various metal oxides, such as Co_3_O_4_, NiO, ZnO, CuO, Cu_2_O, MnO_2_, Fe_2_O_3_, Ag_2_O, SnO_2_, *etc.*^9^ Among various metal oxides explored as a glucose sensors, copper oxide (CuO) is considered to be the best owing to its low fabrication cost, abundance in nature, and favorable electrochemical and catalytic properties.^1,3,10,12,17,25,35^ It is intrinsically a p-type semiconductor with a narrow bandgap of ∼1.2 eV. CuO has also been extensively evaluated for the preparation of electrochemical sensors, photoelectric devices, gas sensing devices and lithium-ion batteries owing to its interesting properties.^16,36–39^ CuO nanostructures, grown on conducting copper foil by a simple method, can be used as low-cost glucose sensors with high sensitivity, fast response and stable detection owing to their superior catalytic properties comparing to those of other metal oxide nanostructures.^9^

Wang *et al.*^40^ synthesized CuO nanofibers and deposited them on an ITO substrate, using electrospinning technique for glucose sensing applications. They achieved high sensitivity of 431.3 μA mM^−1^ cm^−2^ owing to the large specific surface and three-dimensional structure after immobilization. A glucose sensor with flower-like nanostructures of CuO and high sensitivity of 709.53 μA mM^−1^ cm^−2^ has been reported by Wang *et al.*^21^ Zhang *et al.*^10^ have used CuO NWs, synthesized using a facile two-step process *via* a wet chemical route, and obtained glucose sensitivity of 648.2 μA mM^−1^ cm^−2^. In another study, Zheng *et al.*^25^ have prepared a glucose sensor using Ag nanoparticles (NPs)/CuO nanofibers on ITO substrate with an enhanced sensitivity of 1347 μA mM^−1^ cm^−2^ owing to Ag NPs. Ni *et al.*^1^ reported preparation of a CuO NWs based glucose sensor by growing CuO NWs directly on a copper foil by a facile two-step process, using a low-cost apparatus *via* a simple, template-free and solution-based method. They observed very high sensitivity of 1886.3 μA mM^−1^ cm^−2^ with a detection limit of 0.05 μM.

Nanocomposites of noble metals and metal-oxides are expected to be good glucose sensing materials owing to superior electrocatalytic performance and high conductivity of noble metals and high catalytic activity of metal oxides.^41^ In view of the above, Li *et al.*^41^ attempted to use Au/CuO nanocauliflower nanocomposites for glucose sensing for the first time. They observed an enhancement in conductivity and accelerated electron transfer, sensitivity and selectivity toward glucose detection of CuO nanostructures in the presence of gold (Au) nanostructure. In view of the above, we have explored Au NPs as co-catalysts on CuO NWs electrode for enhancing the sensitivity of the glucose sensors. The enhanced catalytic properties and high direct electron transfer *via* CuO NWs in the presence of Au NPs owing to large surface-to-volume ratio have been explored, resulting in drastic improvement in sensitivity and linear range of the glucose sensor under study.

## Experimental section

### Materials

Highly pure copper foil (99.9%) was purchased from Alfa Aesar, Thermo Fisher Scientific (India). Ammonium persulfate [(NH_4_)_2_S_2_O_8_], sodium hydroxide (NaOH), hydrochloric acid (HCl), acetone, isopropanol, and malt extract powder were purchased from Merck Life Science Private Limited (India). Glucose, sucrose, and uric acid have been purchased from Sisco Research Laboratories Private Limited (India). All chemicals were of analytical grade and ultra-pure, hence, no further purification was required. Ultra-pure deionized (DI) water (resistivity 18 MΩ cm) was obtained using Merck Millipore system.

### Electrode preparation

#### Preparation of Cu(OH)_2_ NWs electrode

Small squares (5 mm × 5 mm) of copper foil were used as the starting materials for electrodes. These copper foils were cleaned ultrasonically in DI water, HCl, acetone, and isopropanol sequentially. The foils were immersed in a solution consisting of 80 μl of 10 M NaOH, 180 μl of H_2_O, and 40 μl of 1 M (NH_4_)_2_S_2_O_8_. After 30 minutes, the foils were taken out of solution and dried in air. A deep blue film formed, which was nothing but Cu(OH)_2_ NWs on Cu foil:^1^

1 Cu + 2NaOH + (NH_4_)_2_S_2_O_8_ → Cu(OH)_2_ + Na_2_SO_4_ + 2NH_3_ + 2H_2_O

#### Preparation of CuO NWs electrode

The obtained Cu(OH)_2_ NWs grown on Cu foils were kept on alumina boat and placed inside the furnace with the flow of argon gas for 30 minutes. After 30 minutes the flow of argon gas was stopped and the foils were heated to 120 °C for 3 hours. Further, the foils were heated to 180 °C for 2 hours to promote crystallization. The blue film turned black and became CuO nanowire:^1^

2 Cu(OH)_2_ → CuO + H_2_O

#### Preparation of gold NPs modified CuO NWs electrode

The obtained black coloured CuO NWs were first rinsed with a solution of 8 mg chloroauric acid (HAuCl_4_) in 3 ml DI water for 10 minutes. Then these foils were immersed into solution of 9 mg sodium borohydride (NaBH_4_) in 3 ml methanol. Finally, the foils were rinsed with DI water for 2 minutes to obtain gold NPs (GNPs) modified CuO NWs electrodes. Formation of gold nanoparticles is illustrated by the following reactions:^42,43^

3 NaBH_4_ + 4CH_3_OH ⇔ NaB(OCH_3_)_4_ + 4H_2_

4 4HAuCl_4_ + 3NaBH_4_ → 4Au + 6H_2_ + 3NaCl + 3BCl_3_ + 4HCl

#### Electrode characterization and electrochemical set-up

**Fig. 1:**
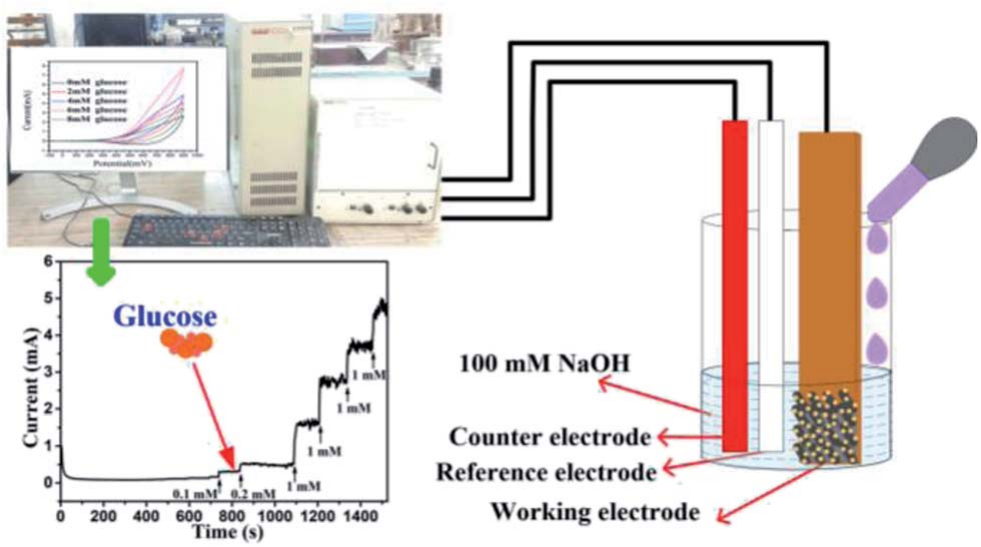
Electrochemical set-up for detection of glucose.

Crystal structure and phase of the electrodes were determined using X-ray diffraction (XRD) (RIGAKU-Smart XDMAX, PC-20, 18 kW Cu rotating anode, Rigaku, Tokyo). Surface morphologies of different electrodes, copper foil, Cu(OH)_2_ NWs, CuO NWs, and Au NPs modified CuO NWs were investigated using scanning electron microscopy (SEM) (Model: EVO MA 15/18 from Carl Zeiss Microscopy Ltd., UK). The Au NPs modified CuO NWs were examined by high-resolution transmission electron microscopy (HRTEM) and scanning transmission electron microscopy (STEM) (FEI G2 T20 STWIN). Electrochemical measurements were performed in a 3-electrode electrochemical cell configuration (BAS100B electrochemical workstation) with 4 CuO based electrodes as working electrodes, a platinum wire as a counter electrode, and Ag/AgCl as a reference electrode as shown in Fig. 1. A solution of 0.1 M NaOH in DI water was used as an electrolyte for the cyclic voltammetry measurements at room temperature.

## Results and discussion

### Characterization of electrodes

**Fig. 2:**
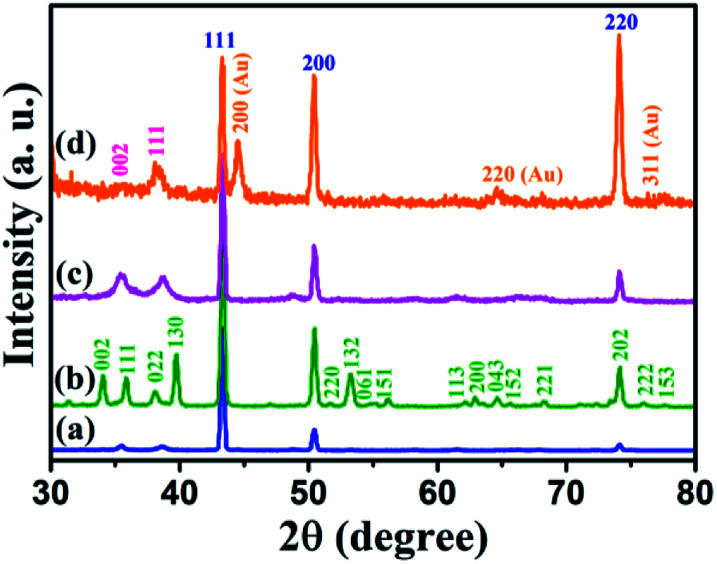
XRD patterns of (a) Cu electrode, (b) Cu(OH)_2_ NWs electrode, (c) CuO NWs electrode, and (d) Au NPs modified CuO NWs electrode.

The XRD patterns of Cu foil, Cu(OH)_2_ NWs, CuO NWs, and Au NPs modified CuO NWs were compared with those of standard JCPDS file numbers 04-0836, 35-0505, 80-1917, and 04-0784 of Cu, Cu(OH)_2_, CuO, and Au, respectively, as shown in Fig. 2. The crystalline sharp peaks found at 43.37 degree, 50.61 degree, and 74.27 degree correspond to (111), (200) and (220) of FCC Cu, confirming the composition of the foil. The others 2 minor peaks at 35.39 degree and 38.65 degree are due to impurities of native oxide of copper. The XRD pattern of Cu(OH)_2_ NWs sample, shown in Fig. 2(b), matches well with the orthorhombic phase (JCPDS card # 35-0505), thereby confirming the successful growth of Cu(OH)_2_ NWs on the Cu foil. Heat-induced dehydroxylation leads to recrystallization and formation of CuO nanowire as evident from the XRD plot (JCPDS card # 80-1917), shown in Fig. 2(c). Comparison of the XRD pattern of the Au NPs modified CuO NWs in Fig. 2(d) with those of CuO and Au confirms that the other minor peaks in the XRD pattern of Au NPs modified CuO NWs result from a small amount of Au NPs grown on CuO NWs.

**Fig. 3:**
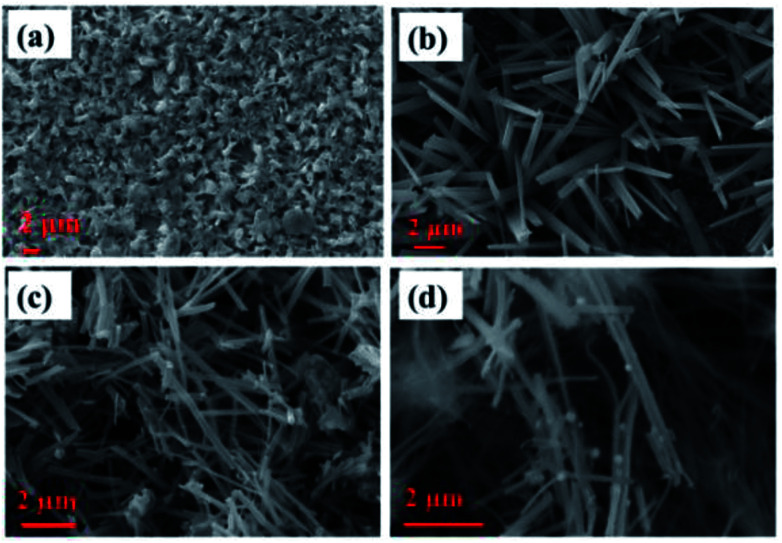
SEM images of (a) Cu electrode, (b) Cu(OH)_2_ NWs electrode, (c) CuO NWs electrode, and (d) Au NPs modified CuO NWs electrode.

Surface morphology images obtained for various electrodes, such as Cu, Cu(OH)_2_ NWs grown on Cu foil, CuO NWs grown on Cu, and Au NPs modified CuO NWs grown on Cu, are shown in Fig. 3(a)–(d). SEM image of the copper foil in Fig. 3(a) shows that the copper foil is coarse. The wire-like morphology of Cu(OH)_2_ with an average diameter of 150–350 nm, uniformly covering the copper foil, is observed in the SEM image shown in Fig. 3(b). SEM image in Fig. 3(c) shows uniformly distributed CuO NWs (of the average diameter of 100–300 nm) on Cu foil. This wide variation in the diameter of CuO NWs may be due to the 2 *in situ* attached NWs which cannot be distinguished by SEM because of its low-resolution limitation. Finally, surface morphology of Au NPs modified CuO NWs under study is shown in Fig. 3(d). SEM image in the figure shows a non-uniform distribution of Au NPs on the CuO NWs.

**Fig. 4:**
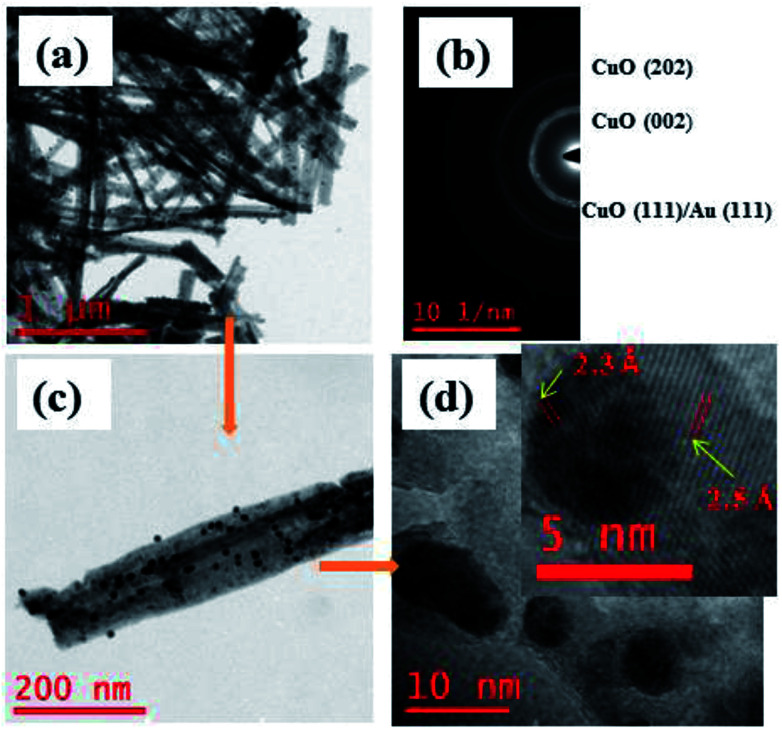
(a) HRTEM images of free-standing Au modified CuO NWs; (b) SAED pattern of selected area; (c) magnified version of a single Au NPs modified CuO NW; (d) further magnified view of Au NPs and CuO NWs (further magnified version is in the inset for *d*-spacing evaluation).

Surface morphology of the Au NPs modified CuO NWs under study was further analyzed by HRTEM, as shown in Fig. 4. The bright field image of Au NPs over freestanding CuO NWs after scrapping from the Cu substrate has been found in Fig. 4(a). The image clearly shows well distributed Au NPs on CuO NWs substrate as small dark dots. The mass-thickness contrast (higher atomic weight of Au as compared to the average atomic weight of Cu and O) is the reason for the dark colour of the Au nanoparticles. Average diameters of the CuO NWs and Au NPs are 75–125 nm and 10–15 nm, respectively. The lengths of the CuO NWs are 1–2 μm. The selected area electron diffraction (SAED) pattern of the Au NPs modified CuO NWs composites from the scanning image area is shown in Fig. 4(b). The diameter of the first 3 circles corresponds to CuO (002), CuO (111)/Au (111), and CuO (202) with *d*-spacing of 2.5 Å, 2.32 Å, and 1.58 Å, respectively. The amount of Au is very small, so it is not possible to confirm the presence Au using SAED. The single nanowire from the magnified version of Fig. 4(a) is shown in Fig. 4(c). It is observed that the Au NPs are well distributed over CuO NWs, as shown in Fig. 4(c). The highly dense distribution of Au NPs on the CuO NWs is observed in our earlier experiment and shown in Fig. S1.† The further magnified image is shown in Fig. 4(d), where the white smoother parts are of CuO NWs and dark dots are of Au NPs. The inset image of Fig. 4(d) shows the lattice spacing measurements for white and dark regions as 2.5 Å and 2.32 Å, respectively, which confirm the presence of CuO (002) and Au (111). The *d*-spacing calculation using HRTEM is shown in Fig. S2 and S3.†

**Fig. 5:**
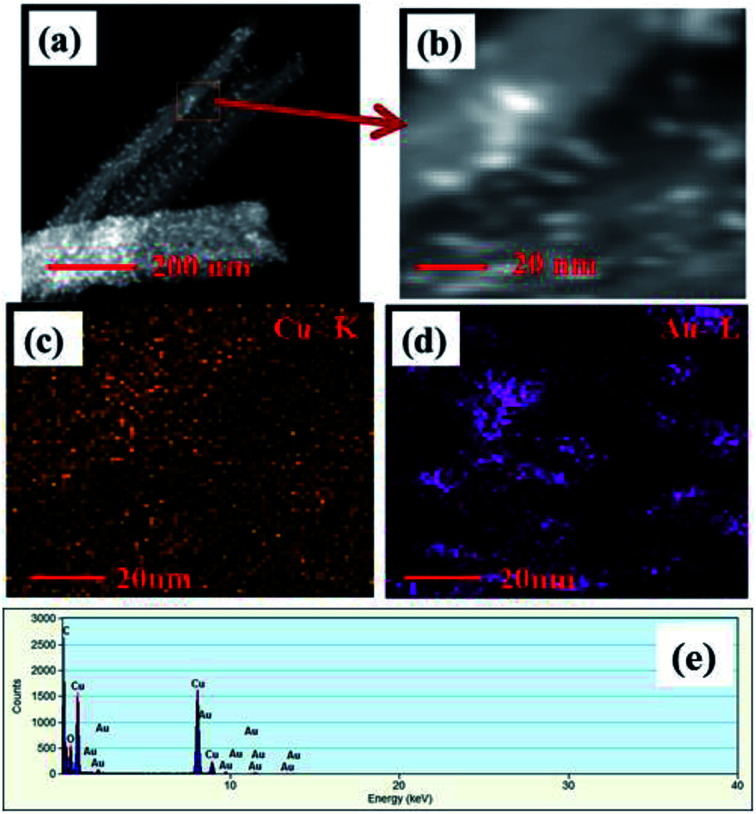
(a) HAADF-STEM images of single free-standing Au modified CuO NWs, (b) magnified view of STEM image of Au NPs and CuO NWs, (c) mapping of CuO NWs, (d) mapping of Au NPs, and (e) EDS spectrum of the selected area.

STEM images of Au NPs modified CuO NWs are shown in Fig. 5. Fig. 5(a) depicts a STEM-HAADF (high angle annular dark field) image of a single CuO NW along with Au NPs. Small white dots are due to the high atomic weight of Au NPs which are easily obtained from Z-contrast using HAADF. Fig. 5(b) is the magnified view of Fig. 5(a) where the distribution of Au NPs over CuO NWs is clearly seen. The yellow dotted region in Fig. 5(c) confirms the presence of CuO NWs, whereas the violet dotted region in Fig. 5(d) confirms the presence of Au NPs. The EDS mapping, shown in Fig. 5(e), confirms the presence of elements such as Cu, O, and Au in the composites. It is easily observed from the EDS mapping that the amount of Au NPs compared to the amount of CuO NWs is very small.

### Electrocatalytic capacity of electrodes towards glucose oxidation

The working electrode CuO NWs get oxidized electrochemically to form Cu(iii) species, such as Cu(OH)^−^_4_ or CuOOH:^44^5
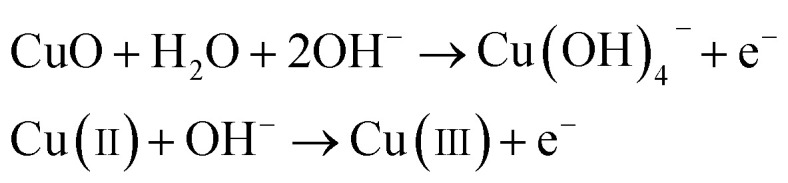


It was reported earlier that oxidation of glucose is enhanced in the presence of copper and its compounds owing to their strong catalytic capability.^45^ The following chemical reactions illustrate oxidation of glucose in the presence of Cu(iii):^44,46,47^6
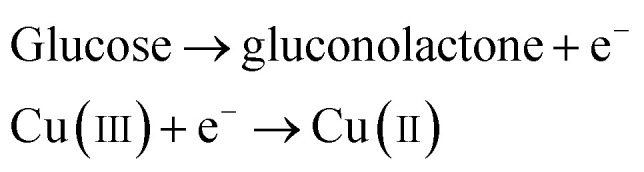
7
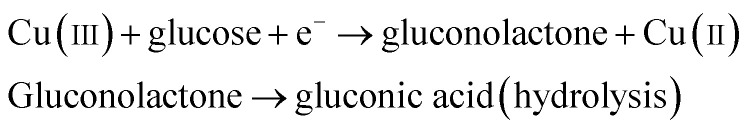


**Fig. 6:**
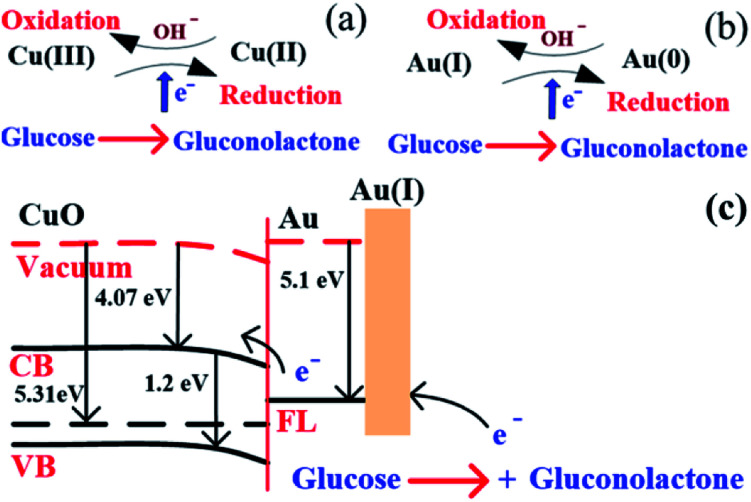
(a) Glucose detection mechanism in CuO, (b) glucose detection mechanism in Au, and (c) glucose detection mechanism in CuO NWs/AuNP structure under applied potential.

The above reaction mechanism is also shown in Fig. 6(a). Similarly, the Au NPs modified CuO NWs electrode is present in the reaction mechanism, as shown in Fig. 6(b). Thus, the overall oxidation of glucose has been increased. It may also be noted that the glucose oxidation reaction is not spontaneous and it is catalysed by Cu(iii) and/or Au(i). These 2 species formed *in situ via* electrochemical hydrolysis process: Cu(ii) → Cu(iii) + e^−^ (redox potential −2.41 V) and Au(0) → Au(i) + e^−^ (redox potential −1.83 V). This step is followed by glucose oxidation, which is oxidized to gluconolactone (redox potential −0.32 V). The above formation of gluconolactone is facilitated by Cu(iii) and/or Au(i). Even though gluconolactone formation is more feasible for Cu(iii) mediated reaction, as apparent from the redox potential value, the rate determining step of the overall reaction is the formation of Cu(iii) or Au(i). Since the formation of Au(i) is more feasible than formation of Cu(iii), Au exhibits higher glucose oxidation rate. Au and CuO interface plays a very important role in this overall electrochemical oxidation process as it impacts the charge transport from the Au NPs to Cu foil through CuO NWs. The negligible Au/CuO barrier, as seen in Fig. 6(c), ensures that impediment in the carrier transport is minimum. Apart from these catalytic differences between CuO and Au, the hybrid structure of the electrode is beneficial for oxidation of glucose because of the enhanced surface area owing to the 3D electrode structure, low cost of electrode using a very small amount of Au NPs, and a large increase in the number of reactive centers owing to larger surface to volume ratio of Au NPs. The complete process of glucose oxidation and charge transfer is described in Fig. 6. When glucose is introduced into 0.1 M NaOH solution, it gets oxidized in the presence of CuO/Au and this electrochemical reaction releases an electron. The released electron moves to the conduction band (CB) of CuO NWs *via* Au NPs. Further, this electron moves in the Au–CuO–Cu electrode network, resulting in a solid-state charge transfer, as shown in Fig. 6(c).

**Fig. 7:**
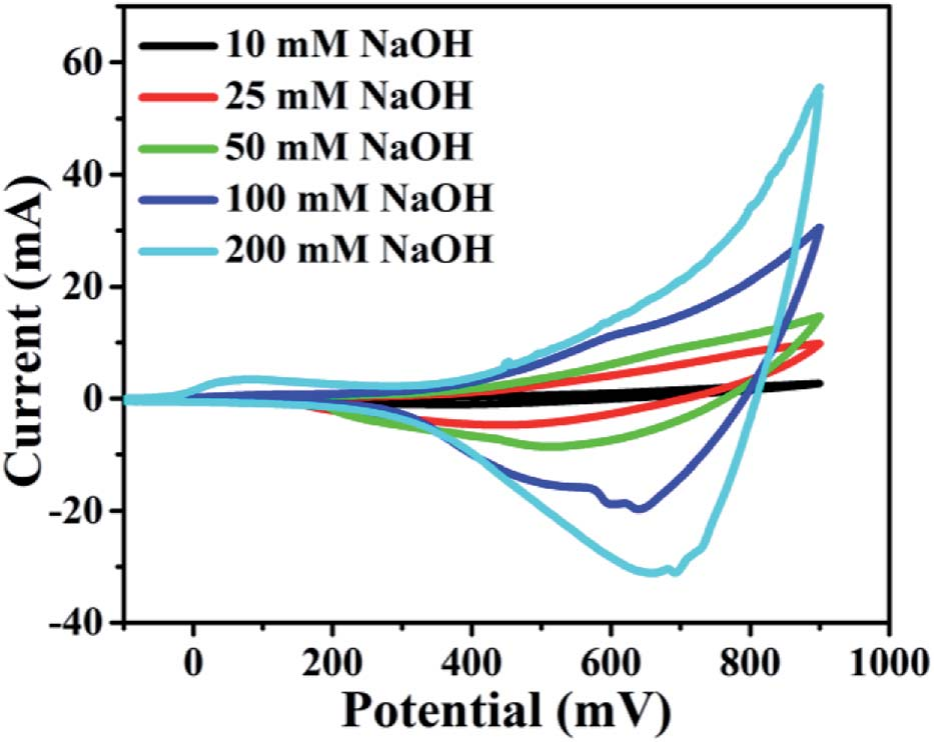
Electrocatalytic effect of different concentrations of NaOH: 10 mM, 25 mM, 50 mM, 100 mM, and 200 mM with scan rate 100 mV s^−1^.

**Fig. 8:**
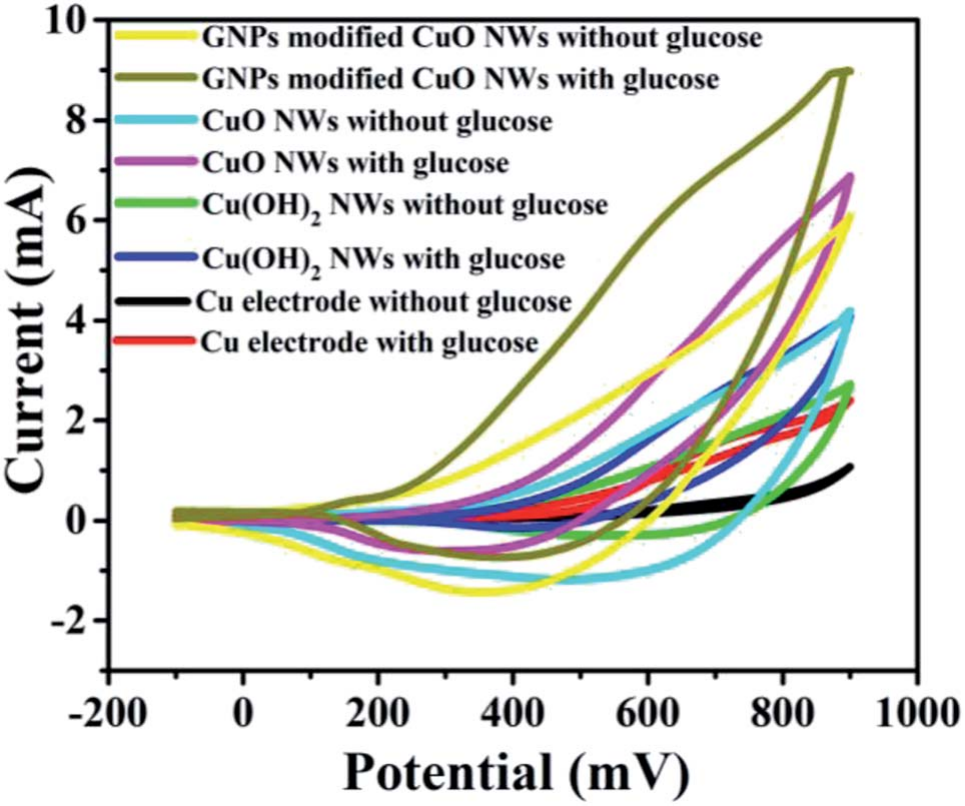
*C*–*V* plots of Cu, Cu(OH)_2_ NWs, CuO NWs, and Au NPs modified CuO NWs electrodes at 0 mM and 4 mM of glucose.

**Fig. 9:**
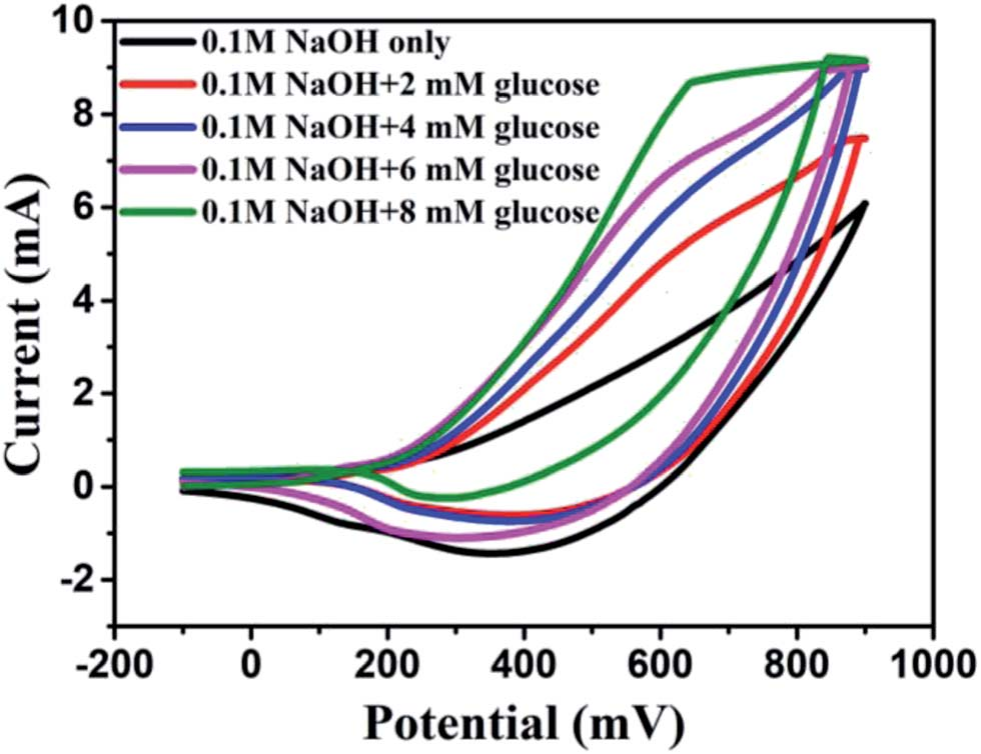
*C*–*V* plots of Au modified CuO NWs electrode at different concentrations of glucose from 0 mM to 8 mM.

**Fig. 10:**
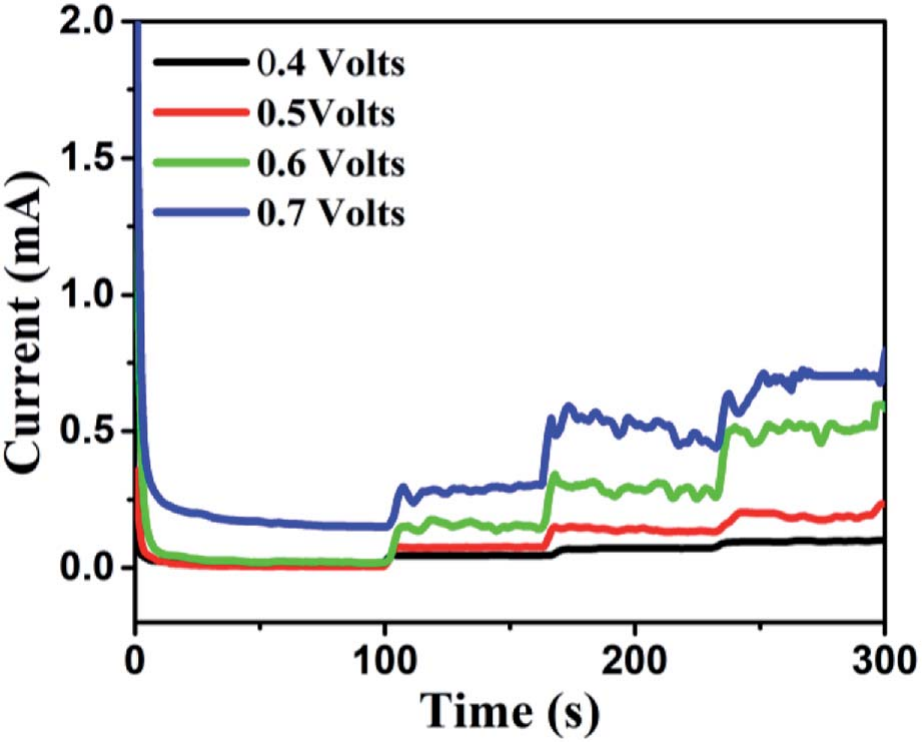
Transient current responses of Au NPs modified CuO NWs at different working potentials and successive additions of 0.25 mM glucose into 0.1 M NaOH solution.

It was found that CuO NWs have the highest electrocatalytic performance when 0.1 M NaOH solution was used as electrolyte, similar to results in ref. 1 We have also observed that similar to CuO NWs electrode, Au NPs modified CuO NWs have the highest electrocatalytic ability when 0.1 M NaOH is used as an electrolyte, as shown in Fig. 7. It is also observed that the oxidation current is a function of scan rate, as shown in Fig. S4 and S5.† Therefore, an optimized scan rate of 50 mV s^−1^ was used in this study. In view of the above, the electrocatalytic behaviours of various electrodes have been investigated in the 0.1 M NaOH electrolyte solution in the potential range from −0.1 to 0.9 V. A fixed amount of 4 mM of glucose is used for all electrodes. It was found that the oxidation current increases with the amount of glucose in the electrolyte for all the electrodes, as shown in Fig. 8. The amount of change in oxidation current due to the presence of glucose in Cu(OH)_2_ NWs is 1.11 times higher than that of bare Cu electrode. This increase in oxidation current is due to the increase in the effective surface to volume ratio for Cu(OH)_2_. On the other hand, the change in oxidation current of CuO electrode is 1.77 times higher than that of Cu(OH)_2_ electrode. A further 2.86 times enhancement in the change in oxidation current is observed when Au NPs modified CuO NWs electrode is used as the working electrode. This enhancement in oxidation current is attributed to the gradual increase in the surface to volume ratio in Cu (OH)_2_ NWs, CuO NWs and Au NPs modified CuO NWs. We have also investigated the current response of the Au NPs modified CuO NWs electrode to different concentrations of glucose varying from 0 mM to 8 mM, dropped into the electrolyte. We have observed a proportional increment in the current with the increase in glucose concentration, as shown in Fig. 9. The transient current response is also investigated for a potential range from 0.4 V to 0.7 V, obtained by successive addition of 0.25 mM of glucose to 0.1 M NaOH of electrolyte. The highest response sensitivity to glucose is observed at 0.6 V, as shown in Fig. 10. The Au NPs modified CuO NWs electrode was also used for measuring higher concentration of glucose, as shown in Fig. S6 and S7.†

### Transient current response to glucose at a working potential of 0.6 V

**Fig. 11:**
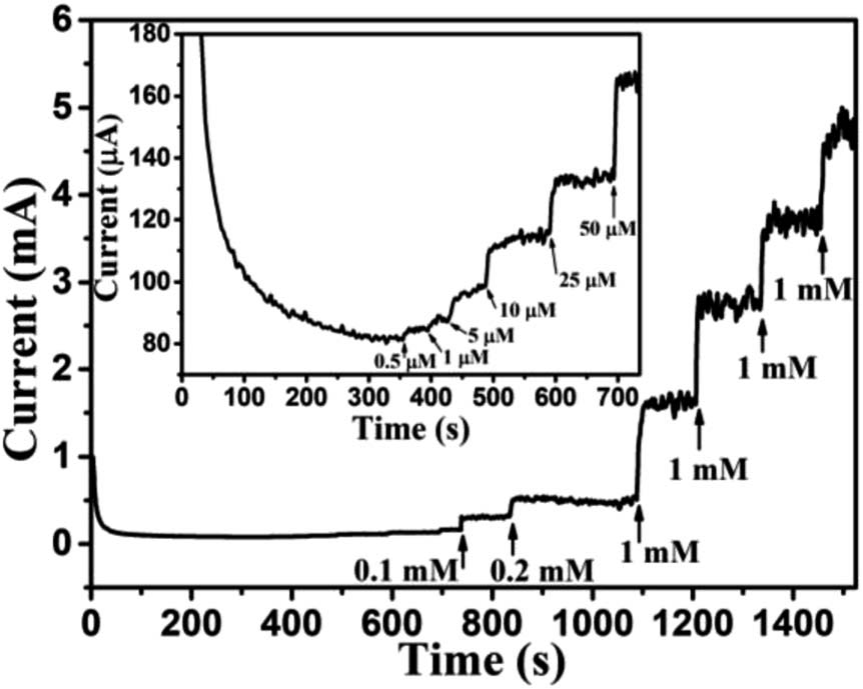
Transient current response of Au NPs modified CuO NWs at 0.6 V with successive addition of glucose (0.1 mM, 0.2 mM, 1 mM, 1 mM, 1 mM, and 1 mM). Transient response with a low concentration of glucose (0.5 μM, 1 μM, 5 μM, 10 μM, 25 μM, and 50 μM) is shown in the inset of the figure.

**Fig. 12:**
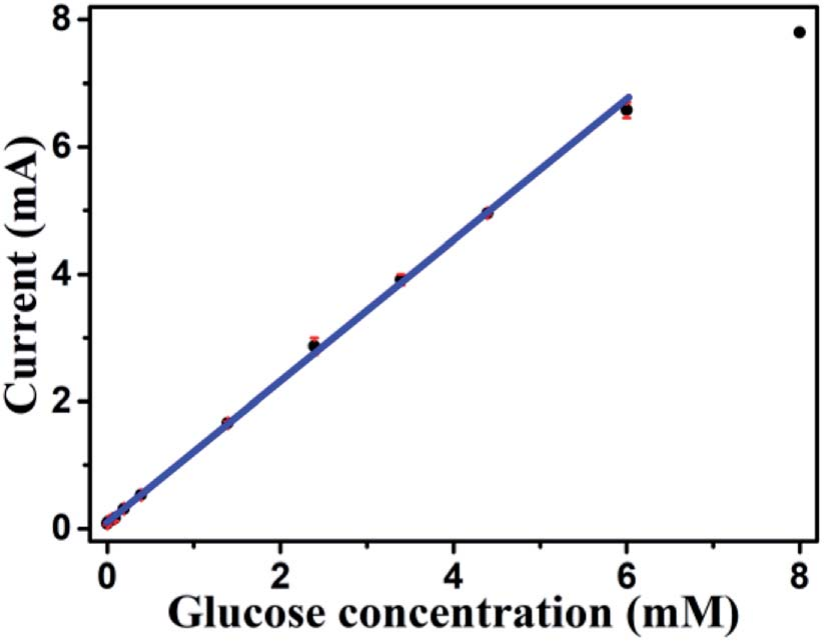
Sensitivity plot of Au modified CuO NWs electrode for the different concentrations of glucose from 0.5 μM to 8 mM.

Based on results shown in Fig. 10, the Au NPs modified CuO NWs electrodes were used as working electrodes for electrochemical measurements at an applied bias of 0.6 V. The known amount of glucose was successively added to the 0.1 M NaOH electrolyte after obtaining a saturated current. As the glucose concentration was increased from 0 mM to 8 mM in the electrolyte solution, a significant increase in the current was observed, as shown in Fig. 11. The sensor had a very fast response time, which was calculated as time to reach 10% to 90% of the sensor current; the average response time was found to be ∼5 s. The sensor was also observed to maintain a good linearity from 0.5 μM to 5.9 mM (*R*^2^ = 0.999), as shown in Fig. 12. The linearity was found to be better than that of the other reported glucose sensors using only CuO nanostructure as electrode material.^1,17,40,44^ The detection limit and sensitivity were found to be 0.5 μM and 4398.8 μA mM^−1^ cm^−2^, respectively, which are better than the values reported by others.^1,17,40,44^ The various sensor parameters were compared with those of the previously reported CuO based glucose sensors and are listed in Table 1. It can be seen from the table that our results are superior in terms of a combination of sensitivity, linear range and detection limit. We have observed that the linear ranges for glucose levels depend on NaOH concentration (pH level).^1,3,41,44,48,56^ It is seen that the lower linear range is found for 0.1 M NaOH solution,^1,44,48,56^ whereas higher linear range is found for 1 M NaOH solution.^3,41^ We have also obtained a similar trend for the linear range of 5.9 mM for 0.1 M NaOH solution. The combination of results of the large linear range, the sensitivity of 4398.8 μA mM^−1^ cm^−2^ and the detection limit of 0.5 μM is superior to the one reported previously.^1,44,48,56^ The broad linear range (up to 50 mM) is obtained for 1 M NaOH solution, as shown in S7 of ESI.†

**Table 1:** Comparison of CuO nano-materials-based glucose sensors

Type of electrodes	Detection limit (μM)	Sensitivity (μA mM^−1^ cm^−2^)	Linear range (mM)	References
CuO NWs	0.05	1886.3	0.002–3.56	1
CuO NWs/Cu foam	0.3	2217.4	0.001–18.8	3
CuO nanosphere	1	404.53	0–2.55	17
CuO flowers	4	709.52	0.004–8	21
Ag NPs/CuO nanofibers	0.052	1347	0.0005–0.5	25
CuO nanofibers	0.8	431.3	0.006–2.5	40
CuO nanoplatelets	0.5	3490.7	Up to 0.8	44
CuO nanourchins	1.97	1634	Up to 5	48
Flower-like CuO	1.2	5368	0.005–1.6	49
Sandwich-structured CuO	1	5342.8	0–3.2	50
CuO/CFF	0.27	6476	0.0003–0.96	51
CuO nanothorn arrays	0.275	5984.3	0.0002–2	52
CuO nanostructure	49	1620	Up to 4	53
CuO/TiO_2_ nanotube	1	79.9	0–2	54
CuO-ZnOnanorods	0.4	2961.7	Up to 8.45	55
Au/CuO nanocauliflowers	0.3	708.7	0.001–30	41
Au/CuO nanohybrids	2.8	374	Up to 12	56
Au NPs modified CuO NWs	0.5	4398.8	0.0005–5.9	This work

### Selectivity, reproducibility, repeatability, and stability of Au NPs modified CuO NWs electrode

**Fig. 13:**
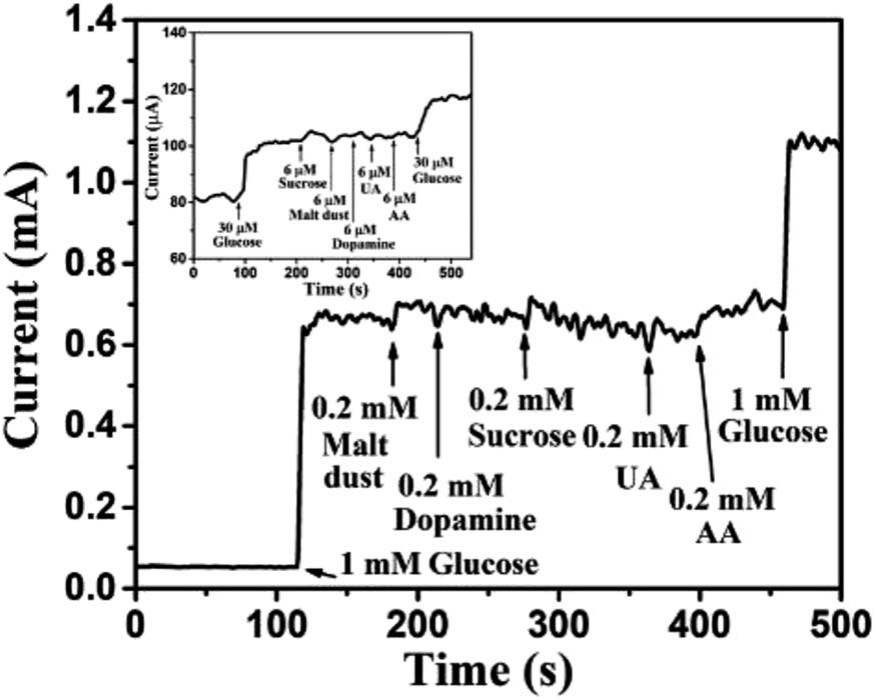
Transient response of Au NPs modified CuO NWs electrode at 0.6 V with successive addition of 1 mM glucose, 0.2 mM malt dust, 0.2 mM DA, 0.2 mM sucrose, 0.2 mM UA, 0.2 mM AA, and 1 mM glucose. The selectivity for low concentration of glucose (30 μM) over 6 μM of sucrose, malt dust, DA, UA and AA is shown in the inset.

Human blood serum contains glucose along with various interfering elements, such as sucrose, malt dust, uric acid (UA), ascorbic acid (AA), and dopamine (DA). However, concentration of glucose in human blood serum is very high, about 30 times higher than concentration of the aforementioned interfering elements.^1,57^ Thus, most researchers^1,3,34,55,57^ have used glucose concentrations of 5–10 times higher than the concentration of other interfering elements for the practical analysis of human blood serum. In view of the above, 1 mM glucose was selected for 0.2 mM of each of the interfering elements, such as sucrose, malt dust, UA, AA, and DA for investigating the selectivity of the sensor, as shown in Fig. 13. The glucose concentration as low as 30 μM has also been chosen for the selectivity study, as shown in the inset of Fig. 13. Significantly larger currents for glucose than for the interfering components demonstrate high selectivity of our proposed glucose sensor, investigated in this article. The change in current for glucose was observed to be significantly higher as compared to that of other interfering components owing to the enhanced oxidation of glucose as compared to other interfering components.^44^ Reproducibility was investigated using 5 different Au NPs modified CuO NWs electrodes. It was found that the results obtained with these electrodes were in good agreement and have an error of ±2.5% only. To test the repeatability of the proposed electrode, the electrochemical measurements were carried out 5 times using individual electrode and the results were repetitive with a slight error of <±2%. The proposed Au NPs modified CuO NWs electrode was also tested over 10 days with an interval of 2 days, and relatively better stability results were obtained as compared to those obtained by using the bare CuO NWs electrode.

### Measurement of glucose in human serum samples

**Fig. 14:**
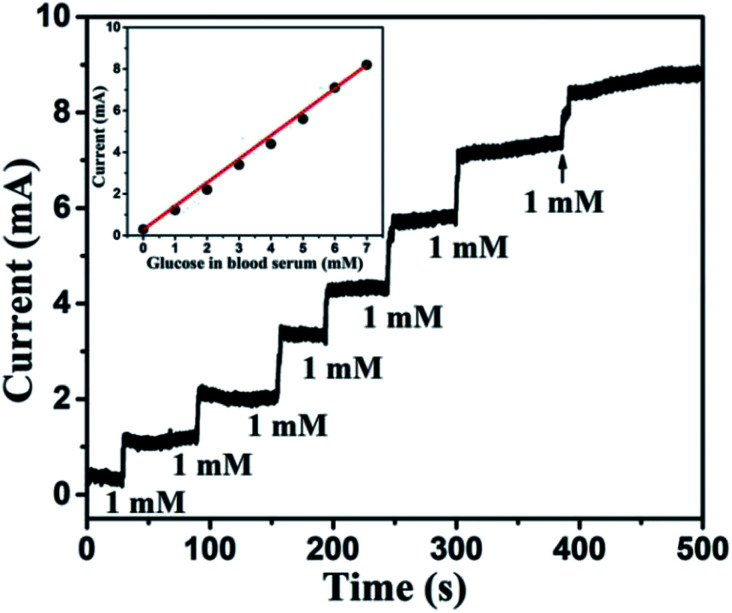
Transient response of Au NPs modified CuO NWs electrode at 0.6 V with successive addition of human blood serum corresponding to 1 mM glucose. The nearly linear glucose response of blood serum is shown in the inset.

To probe the practical relevance of the viability of this reported glucose sensor, it was also tested in human blood serum, containing glucose which can be detected. The blood samples were collected with the help of the university students' health centre (Sir Sundar Lal Hospital, Banaras Hindu University, Varanasi, India). The blood samples of a healthy person were donated by someone from our research group. About 100 μl blood was added to 9.90 ml of 0.1 M NaOH and the corresponding oxidation current response was measured at 0.6 V. The concentration of sugar in a blood sample was evaluated using the reference current through calibration curve obtained from the electrochemical measurement. The obtained transient response of the sensor towards human blood serum and linearity plot are shown in Fig. 14. The slight hazy and the higher current response was obtained for human blood serum, owing to the interference of various components of blood serum. The glucose response was nearly linear (correlation coefficient less than 0.99), and the glucose level of 130 mg dl^−1^ (>7 mM) can be measured efficiently. We compared these results with standard results provided by the health centre. Each sample was measured 3 times and data are listed in Table 2. We can easily match our results with standard results. Glucose concentration can also be determined in the diluted serum samples. Since the concentration of glucose is reduced when the serum is diluted, the amount of glucose in the diluted serum samples, determined by using the proposed method, is required to be multiplied by the inverse factor of serum dilution to obtain the desired result. Thus, the proposed sensor is very effective for glucose detection.

**Table 2:** Detection of glucose in human blood samples^a^

Sample	Glucose concentration (mM)		Recovery (%)	RSD *X* (%)
	Spectrophotometric method (provided by the health centre)	Proposed method		
1	6.178	6.13	99.22	0.5
2	5.238	5.35	97.90	1.5
3	4.741	4.91	96.56	5.2

a *X* = Relative standard deviation (RSD) of 3 measurements.

## Conclusions

A non-enzymatic easily constructed glucose sensor is reported in this article. Gold nanoparticles (Au NPs) have been deposited on CuO NWs electrode using *in situ* chemical reaction. These Au NPs on the surface of CuO NWs increase the effective surface to volume ratio of the electrode, which in turn increases the catalytic capability of the Au NPs modified CuO NWs significantly as compared to that of the bare CuO NWs. As consequence, the oxidation and reduction properties of the Au NPs modified CuO NWs are improved drastically. The enhanced aforementioned properties of Au NPs modified CuO NWs electrode have been explored for enhancing the detection capability of the CuO based glucose sensors. The Au NPs modified CuO NWs based glucose sensor under study in 0.1 M NaOH solution exhibits sensitivity of 4398.8 μA mM^−1^ cm^−2^ with the maximum linearity upto 5.9 mM, which is a better result than the ones previously reported for the CuO based glucose sensors. The extended linearity range upto 50 mM of glucose is achieved at the cost of increased basicity of electrolyte to 1 M NaOH. Finally, the proposed glucose sensor is used for measuring the amount of glucose in human blood in a practical setting. The results agree well with the practical measurements for glucose in the human blood, carried out in the pathological laboratory. Combination of higher sensitivity and low detection limit along with extremely low fabrication cost of this sensor makes it also suitable for non-invasive detection of glucose through saliva and urine.

## Conflicts of interest

There are no conflicts of interest.

## Supplementary Material

RA-009-C8RA07516F-s001
