# Supplementary material for: Superficial fabrication of gold nanoparticles modified CuO nanowires electrode for non-enzymatic glucose detection
Source: RSC Adv. 2019 Jan 14;9(4):1772–81. doi: 10.1039/c8ra07516f (PMC9059820; doi:10.1039/c8ra07516f)
Supplement: RA-009-C8RA07516F-s001 [file RA-009-C8RA07516F-s001.pdf]

Electronic Supplementary Material (ESI) for RSC Advances.
This journal is © The Royal Society of Chemistry 2018

Supporting Materials

Superficial fabrication of gold nanoparticles modified CuO nanowires electrode for non-enzymatic glucose detection

Ashwini Kumar Mishra[1], Bratindranath Mukherjee[2], Amit Kumar[1], Deepak Kumar Jarwal[1], Smrity Ratan[1], Chandan Kumar[1], and Satyabrata Jit[1]*

[1]Department of Electronics Engineering, [2]Department of Metallurgical Engineering

Indian Institute of Technology (Banaras Hindu University), Varanasi-221005, India

E-mail: *sjit.ece@iitbhu.ac.in

Index

S1: HRTEM image of Au NPs modified CuO NWs …………………………………... (2)

S2: Calculation of d-spacing for CuO NWs ………………………..........…………..... (2)

S3: Calculation of d-spacing for Au NPs ……………………………………………..... (3)

S4: CV curve in 1 M NaOH solution at various scan rate ……………………………... (3)

S5: Oxidation current versus scan rate in 1 M NaOH solution ………………………... (4)

S6: CV curve for higher glucose concentration ……………………………………….. (4)

S7: Linearity range for higher glucose concentration …………………………………. (5)

The Au NPs modified CuO NWs with highly dense Au NPs is shown in Fig. S1. This structure could be considered for a higher linearity range. The inter-spatial distance for CuO NWs is calculated using HRTEM as shown in Fig. S2. The inter-spatial distance for Au NPs is calculated using HRTEM as shown in Fig. S3.

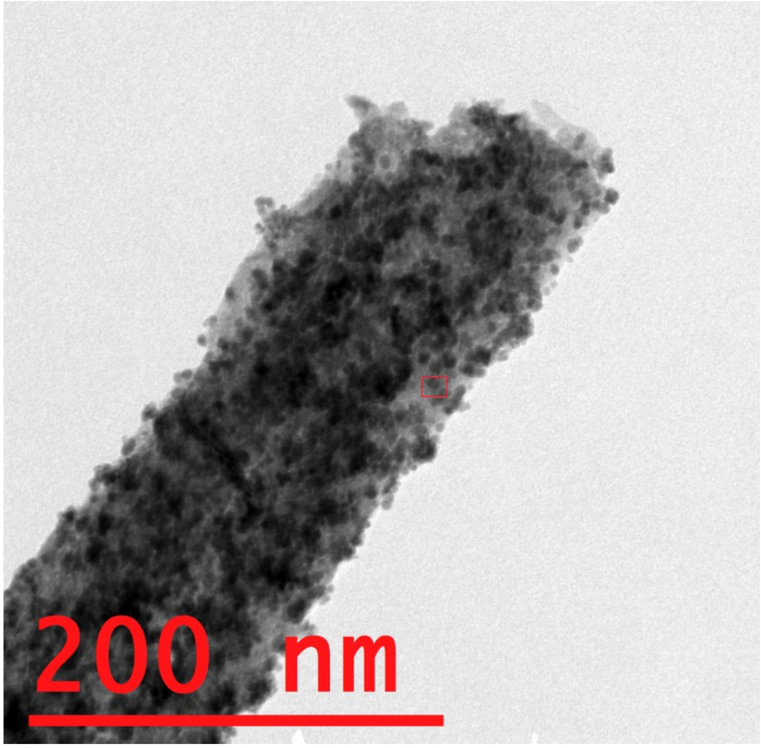

Fig. S1. HRTEM image of Au NPs modified CuO NWs.

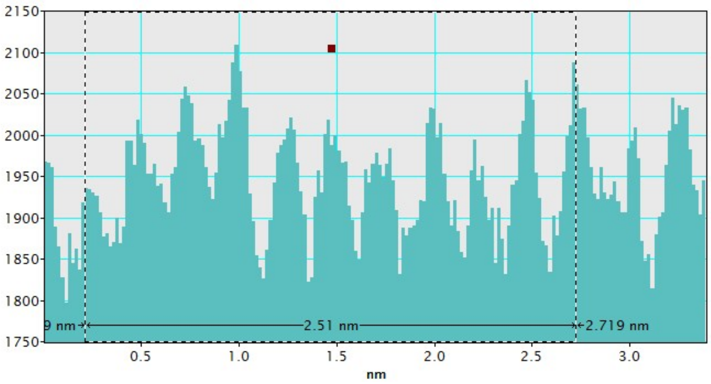

.

Fig. S2. Calculation of d-spacing for CuO NWs.

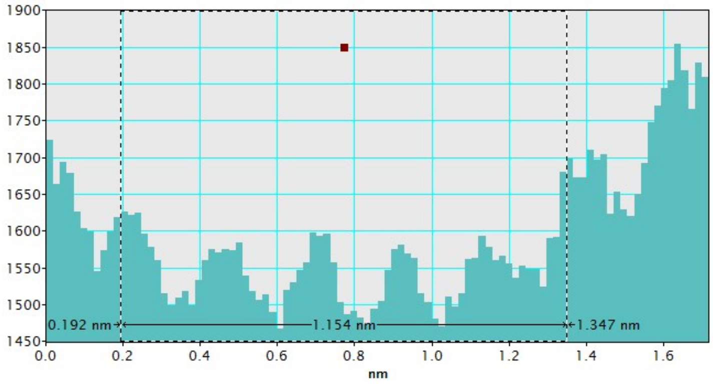

Fig. S3. Calculation of d-spacing for Au NPs.

The cyclic voltammetry (CV) measurement is carried out at various scan rate from 20 mV/s to 200 mV/s in 1 M NaOH solution and 3 mM glucose as shown in Fig. S4. It is found that the oxidation current is increased with the increase in scan rate. This increment in oxidation current is proportional to the scan current as obtained by linear fitting as shown in Fig. S5.

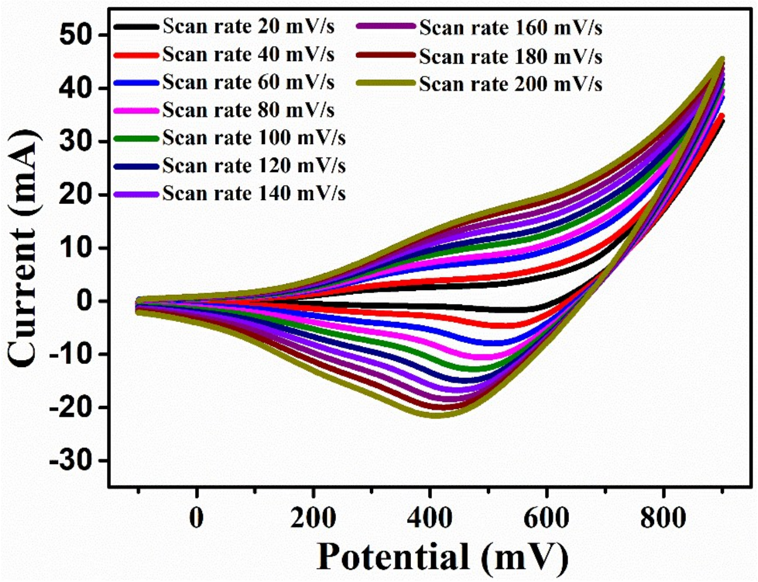

Fig. S4. CV curve in 1 M NaOH solution at various scan rate

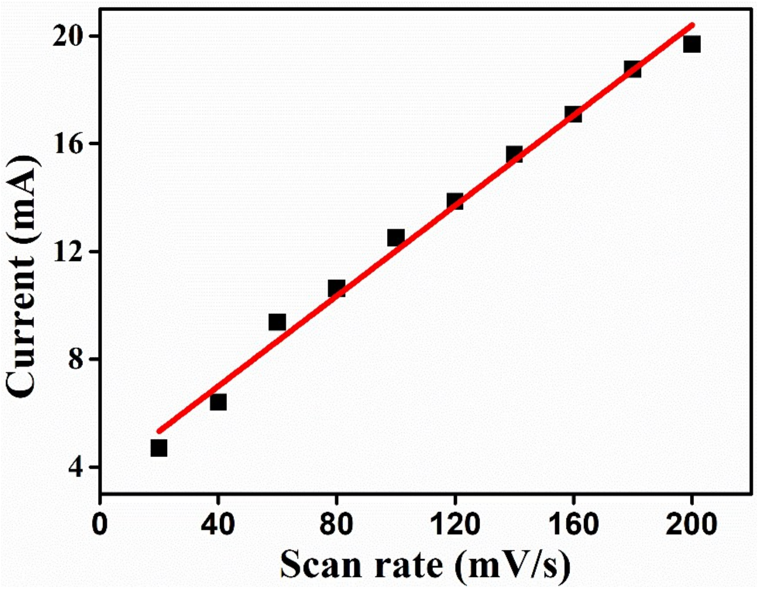

Fig. S5. Oxidation current versus scan rate in 1 M NaOH solution.

The higher glucose concentration is also measured in 1 M NaOH solution using Au modified CuO NWs electrode. The obtained CV curve is shown in Fig. S6. The linearity curve for oxidation current versus glucose concentration at a potential of 0.6 V is drawn in Fig. S7. A broad linearity range (upto 50 mM) is observed in Fig. S7.

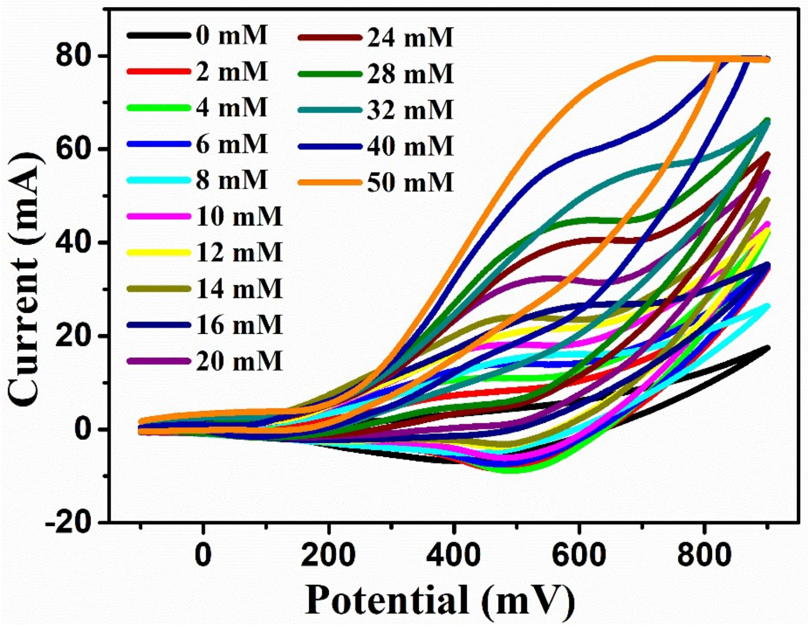

Fig. S6. CV curve for higher glucose concentration.

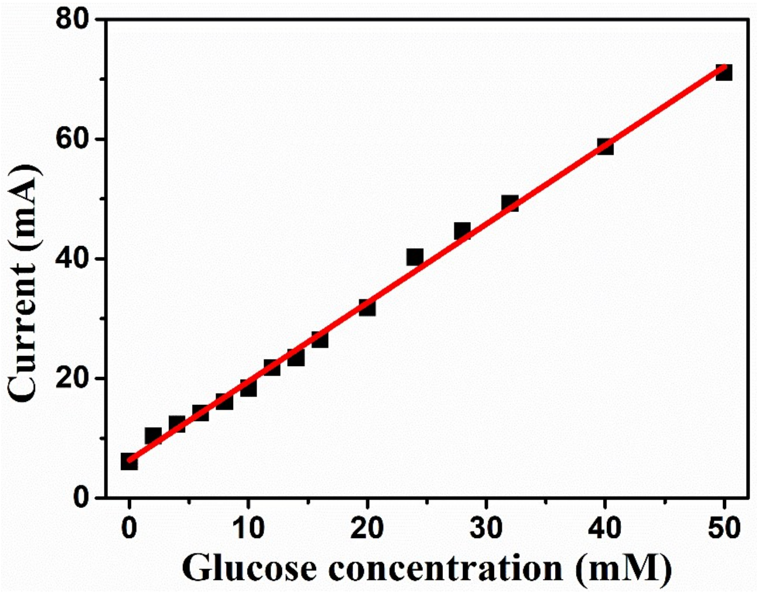

Fig. S7. Linearity range for higher glucose concentration.
